# Postdigital Resonance

**DOI:** 10.1007/s42438-024-00516-x

**Published:** 2024-10-08

**Authors:** Lawrence Wilde, Charles White, Petar Jandrić

**Affiliations:** 1 Institute of Music at Faculty II: Education · Architecture · Arts, University of Siegen, Siegen, Germany; 2 Max Weber Centre for Advanced Cultural and Social Studies at the University of Erfurt, Erfurt, Germany; 3 University of Graz, Graz, Austria; 4 Zagreb University of Applied Sciences, Zagreb, Croatia

**Keywords:** Postdigital Resonance, Co-Presence, Resonance Theory, Human-Technology Entanglement, Datafication

## Introduction

As the boundaries between the digital and the analog become increasingly blurred, our experiences are pluralized and datafied in a web of constant connectivity. This web fundamentally transforms our perception of the world, altering ‘our relationship to time and space, to other people, to the objects around us, and ultimately to ourselves, to our body and our mental dispositions’ (Rosa 2019: 1). Hartmut Rosa (2019: 39–40) presents three ways of relating to the world or the three axes of resonance: (1) social, (2) material, and (3) existential. In an increasingly technology-driven reality, is there a need for a postdigital axis of resonance? Given that ‘the indivisibility of the digital with many other aspects of contemporary life is a foundational principle of postdigital research’ (Knox 2024: 2), it is essential to explore how our experiences are inherently shaped by digital entanglements. As we navigate these complexities, it is crucial that ‘the concept of the postdigital must remain a common good’ (Jandrić et al. 2023: 7), emphasizing the need for socially relevant research that contributes to improving the quality of life.

Consider the following scenario at a café: a person is immersed in simultaneous layers of reality, and each layer pulls them into distinct but interconnected dimensions of existence. This individual navigates through datafied physical and online interactions that define the multidimensionality of our postdigital lives. This person is not present in a single space; they exist simultaneously across multiple spaces and times, with each analog and digital layer (trans)forming their relationship to the world. In this reality, everything from personal experiences and memories to friendships and other human connections is channelled through technology, often leaving traces of data. This individual’s concurrent presence(s) can be delineated as follows:

- **Presence 1:** The individual is physically seated in a lively café in an urban landscape, surrounded by people, sights, sounds, and smells of the physical world. This physical setting provides the foundational reality from which all other interactions stem.
- **Presence 2:** On their laptop, a class is in session via a videoconferencing software with the professor and other students in a hybrid classroom.
- **Presence 3:** Concurrently, by moving the videoconferencing software window out of the way on their computer screen, the individual is immersed in a multiplayer video game, competing with players worldwide. This interaction occurs in a shared virtual environment that is as real in its consequences and engagements as the physical space they occupy.
- **Presence 4:** The gaming experience is not just for personal enjoyment; it is also being streamed live, adding another layer of connectivity.
- **Presence 5:** Integral to the live streaming is the engagement with the viewers’ chat. The person responds to comments and questions in real time, managing a social interaction that is both global and instantaneous.
- **Presence 6:** In the background, through headphones, a rock concert recorded in the 1970s fills their ears. This musical experience transports them to yet another time and space.
- **Presence 7:** Amidst these activities, the individual maintains a text conversation with a family member, occasionally picking up the phone and placing it down.
- **Presence(s): 8, 9, 10 … n**

This scenario is an example of how contemporary individuals connect in a postdigital world.^1^ As Jandrić et al. (2018: 893) explain, ‘we are increasingly no longer in a world where digital technology and media is separate, virtual, “other” to a “natural” human and social life.’ Carvalho and Lamb (2023: 1) further suggest that postdigital alludes ‘to people’s co-presence in multiple spaces, where experiences and interactions with material and digital elements are seen as intertwined’ (see also Carvalho and Freeman 2023; Fawns 2019, 2022; Lamb et al. 2022).

At each presence, there exist social, bodily, material, and digital elements, with each layer of interaction representing a strand of connectivity that is (en)coded into the fabric of our daily life. Giambastiani (2021: 33–34) references Ihde’s (1990) notion of ‘technosphere’ to explain ‘our technological environment … in which we find ourselves and which involves all dimensions of our relations.’ Giambastiani (2021), expanding on both Ihde (1990) and Feenberg (2017), explains that terms such as ‘technosphere’ or ‘ecosystem’ in which our modern environment can be described, ‘should be replaced with *technosystem* because it more correctly describes the texturing “cocoon” in which most of us now live’ (emphasis from the original).

In education, where integration of digital technologies into everyday school life becomes ubiquitous, new concerns about inequality, privacy, and autonomy emerge. Carvalho and Lamb (2023) note that the postdigital stance draws attention to growing disparities and surveillance issues, raising significant ‘questions about spatial privacy.’ This is compounded by the realities described by Jopling (2023), where the future is increasingly dictated by commercial entities wielding sophisticated data mining algorithms. These factors warrant a critical examination of commercialization, datafication, and algorithmization.

These processes contribute to a troubling trend where students are reduced to datasets—identities distilled into bytes and subjected to algorithmic scrutiny. This reduction of human complexity into mere data can lead to future ‘datacism’—potential data-based discrimination practices. Benítez and Romero (2024: 105) write: ‘The social and political implications of how we are going to work in this tech-nological-humanistic convergence is decisive for the future of our societies’ and warn that ‘[t]he trend towards dataism in our society cannot lead us to a *datacism*, where biases and programming failures can cause social or political gaps’ (emphasis from the original).

Our world already sees such forms of discrimination through biased algorithms that affect everything from job applications through college admissions to loan approvals, perpetuating inequalities and marginalizing certain groups based not on race or ethnicity but on how their data is interpreted and valued (Leurs and Shepherd 2017; Van Es and Schäfer 2017). As commercial corporations and tech companies gain more control, the risk of datacism has become a significant social issue. This would necessitate a fundamental (re)evaluation of digital ethics, privacy laws, and the development of mechanisms to ensure that algorithmic decision-making promotes fairness, respects privacy, and upholds fundamental human dignity (Chassignol et al. 2018; Pardo et al. 2019; Hwang et al. 2020; Holmes et al. 2022).

In this context, Hartmut Rosa’s theory of resonance and alienation, as discussed in *Resonance: A Sociology of Our Relationship to the World* (2019), provides a compelling framework for critically examining how ‘technologies mediate the world in such a way that the perception of self, world, and environment changes’ (see Giambastiani 2021; Ihde 1990). Rosa (2019) posits that true resonance requires a transformation in which both the individual and the world—as it is perceived—are altered. This transformation is not only personal but also social, resonating with the tradition of critical pedagogy, which emphasizes collective emancipation and justice.

Jandrić (2023: 3) notes that ‘[p]ostdigital work is strongly informed by the tradition of critical pedagogy with its emphasis on themes related to emancipation and social justice.’ In this sense, the resonance that Rosa (2019) describes extends beyond individual experience to encompass broader social change. Technology plays a dual role in this process; ‘[w]hile technology transforms our experience of our lifeworld, it simultaneously reveals the world in a transformed manner’ (Giambastiani 2021: 34). Focusing on the critical pedagogy aspect of postdigital theory, we can further develop the notion of resonance, adding axes that account for the social and technological transformations central to the postdigital condition.

## *Rosa*’s Three Axes of Resonance and Alienation

As digital technologies permeate all aspects of our lives through the rapid acceleration of technology and pervasive influence of artificial intelligence (AI), Rosa’s (2019) resonance theory can be furthered through the inclusion of postdigital axes of resonance. Rosa identifies three principal axes—social (horizontal), material (diagonal), and existential (vertical)—which provide a framework for analyzing human experiences. Rosa (2019: 92) notes: ‘There can be little doubt that people use screens and digital media to establish contact with others and, in this way secure new relationships to the world.’ Rosa (2019: 92) further articulates that ‘digital media without a doubt have the character of axes of resonance … And so it is no wonder that we jump at every vibration of our smartphone in our pocket, as every incoming message represents a *call from the world*’ (emphasis from the original).

This acknowledgment aligns with the rationale for the inclusion of the postdigital axes as part of the theory of resonance. Rosa’s observation that ‘[i]t is astonishing, then, that all of these resonant signals, large and small, seem to have no lasting effect’ (Rosa 2019: 92), further supports this. While the statement is subjective, it touches on a sentiment many of us can relate to in the postdigital age. It reflects a widespread perception of digital interactions as *transient* and often unsatisfying, hinting at a broader societal alienation and loss of genuine connection(s) to the world. Paradoxically, despite being more interconnected than ever before, we often find ourselves feeling disenchanted, disconnected, overwhelmed, stressed, tired, and drained as we yearn for something deeper amidst the digital inundation.

Rosa’s (2019) observation is subjective because the perceived impact of digital interactions can vary widely among individuals. For some, digital engagement—such as social media interactions, virtual meetings, and online gaming—do indeed foster meaningful connections that have long-lasting effects. These interactions can provide comfort, a sense of community, or valuable information. However, for others, these same interactions might feel superficial or transient, lacking the depth and resonance found in face-to-face encounters. This variability highlights the dynamic nature of such experiences.

Rosa (2019) touches on important social phenomena including burnout and alienation exacerbated by digital media and potentially leading to online apathy. Studies show increased feelings of isolation, depression, and addiction linked to the use of digital technologies (Kraut et al. 1998). For instance, the concept of doomscrolling and the mental health implications associated with digital media consumption point to an objective basis for the lack of resonance in digital technology-mediated experiences. The continuous influx of digital signals—from notifications and alerts to messages and updates—create scenarios where no single interaction has enough time to settle and make a meaningful impact before being overshadowed by the next.

This can lead to what Rosa (2019) describes as a form of addiction: ‘The half-life of feeling assured of digital resonance appears to be inversely proportional to the ever-growing quantity of incoming signals of resonance, leading to a form of increasingly addictive behavior,’ where individuals continuously seek out new digital stimuli, hoping for resonance that rarely lasts. The expansion of resonance theory to include a postdigital axe can contribute to a deeper understanding of how digital media can lead to fragmented presences and influence the sense of fulfillment. A postdigital axe can offer a lens through which to examine the way in which digital technology complicates the sense of resonance in an increasingly digital technology-mediated world.

## A Postdigital Axe of Resonance

Given these undercurrents, the development of a postdigital axe of resonance becomes even more relevant. The notions of resonance and alienation can serve ‘as starting points for further dialogue' and potentially address the need to 'transform postdigital research’ (Veletsianos et al. 2024). As Jandrić (in Veletsianos et al. 2024) explains, ‘[t]his is a hugely important problem, and just as hugely difficult.’ Positioning postdigital research within Rosa’s (2019) theory of resonance and the ‘temporal sociology of social acceleration’ can potentially address Jandrić’s concern of ‘how to make postdigital research more socially relevant’ (Veletsianos et al. 2024). A postdigital axe can lead to more socially relevant postdigital research by having a direct impact on society, addressing problems, needs, and interests that are pertinent to people’s daily lives, communities, and social structures. Postdigital resonance has potential to foster insights, solutions, and understandings, shaping societal conditions, policies, and practices within postdigital cultures.

Establishing a framework for the postdigital axe of resonance can involve expanding and (re)framing concepts such as ‘co-presence,’ ‘entangled human embodiment,’ ‘interdependence of human and non-human agents,’ and ‘placeless places’ within the theory of resonance and alienation (Otrel-Cass 2023; Carvalho and Lamb 2023; Rapanta 2023; Giambastiani 2021; Augé 2008). Establishing a postdigital framework is essential to understand how rapid technological changes influence human connection, identity, and social interaction in increasingly hybrid environments. As digital technologies continue to reshape our realities, expanding Rosa’s (2019) theory to include a postdigital axe offers a framework for understanding how analog, digital, artificial, virtual, and AI domains are becoming entangled in modern life, influencing our interactions, perceptions, and relationships, including but not limited to:

- **Transformation of Space and Time**—postdigital entanglements allow for asynchronous and non-local interactions, altering how we perceive presence and engagement (Lupton 2020; Rode and Stern 2023).
- **New Forms of Community and Identity**—online platforms enable unique communities and identities that might not correspond directly to physical, geographical, or cultural constraints (Al Zidjaly 2019; Baltezarevic et al. 2019).
- **Interactivity and Immersion**—virtual and augmented realities offer immersive experiences that challenge traditional sensory engagements and create new forms of *embodiment* (Otrel-Cass 2023).
- **Digital Materiality**—while digital experiences are mediated through material devices, the nature of digital materiality is distinct as it represents information and interaction, not merely physical form (Otrel-Cass 2023; Davidsen et al. 2023).

Otrel-Cass (2023) argues that ‘postdigital thinking means that online and offline spaces and activities are entangled and inseparable,’ suggesting a realm where the realities converge. Shapiro (2015) further highlights the transformation of our lived experiences through digital embodiment, arguing that our bodies in digital spaces extend beyond physical reality to include various forms of digital embodiment. This indicates a form of engagement that is neither purely social, existential, nor material, but is distinctly shaped by digital media. In these contexts, a postdigital axe can contribute to a deeper understanding of experiences such as identity formation, community engagement, and sensory interactions that occur with and within the digital and often extends or transform the analog.

The development of a postdigital axe of resonance, however, faces similar challenges to that of postdigital research in general. Despite the growing acceptance and application of the term postdigital, resistance persists in various forms. This manifests in debates over the term’s usefulness, its theoretical underpinnings, and its implications for social and material conditions (Sinclair 2023). A significant aspect of resistance involves skepticism toward the term ‘postdigital’ itself. Levinson (2019) critiques the term’s implication of moving ‘beyond’ the digital, arguing that such post-adjectives often lead to misunderstandings and exaggerated claims about the state of technology. Similarly, Selwyn (2023) warns that framing the postdigital as a departure from techno-fascistic imaginaries may overlook the term’s potential for constructive critique. Research also acknowledges that digital technology can often amplify societal inequities and exploitation rather than transcend them (Cormier et al. 2019). These reservations illustrate a broader concern that the term may inadvertently perpetuate the very dichotomies it seeks to transcend.

The resistance is not limited to the term and its implications but also encompasses the theorizing and research surrounding the postdigital. Critics such as Hall (2021) argue against the postdigital’s potential for anti-humanist educational practices and advocate for alternative futures that reject possible dystopian conditions. Additionally, Selwyn (2023) expresses concern about the clarity and coherence of postdigital research, suggesting that its fragmented approach may lead to a lack of unified understanding. The diversity of theoretical lenses applied to postdigital research, from Marxist critiques to Actor Network Theory (ANT), reflects ongoing debates about the term’s theoretical validity and its practical implications (Kuhn and Carrigan 2023; Kuhn et al. 2023). Resistance to the postdigital reveals critical concerns about the term’s application, its theoretical foundations, and its implications for social justice. However, while resistance often challenges the term’s current utility and coherence, it also prompts a deeper examination of its role in addressing contemporary issues and shaping future research. This ongoing dialogue is essential for refining our understanding of the human condition.

To address the resistance and broaden the scope of postdigital research, Rosa’s (2019) theory of resonance can be developed to include a postdigital axe. Rosa emphasizes the importance of authentic and emotionally fulfilling engagements, which can counteract the critiques that the postdigital can risk reinforcing existing inequalities or promoting techno-fascistic tendencies. Rosa’s (2019) focus on resonance rather than mere technological progression addresses concerns raised by Levinson (2019) and Selwyn (2023) by shifting the discourse from abstract post-adjectives to tangible experiences of connection and alienation.

Bridging resonance theory with postdigital research allows for a more critical examination of how digital advancements affect our sense of belonging and agency. The proposed postdigital axe of resonance provides a framework for exploring how digital technologies can either enhance or diminish meaningful interactions, addressing Cormier et al.’s (2019) concerns that digital technologies may amplify societal inequities. By focusing on how individuals *resonate* in and with their environments, researchers can identify and address the gaps and challenges highlighted by previous critiques.

Furthermore, Rosa’s (2019) theory can address the fragmentation in postdigital research by offering a coherent lens through which to analyze various theoretical perspectives. A postdigital axe can lead to a more unified understanding of the postdigital by emphasizing the relational aspects of technology and society, thus contributing to a more socially relevant and integrative field of study. Through this lens, postdigital research can evolve to continue effectively engaging with contemporary issues, bridging theoretical debates and offer practical applications.

## Conclusion

Rosa’s (2019) original axes—social, material, and existential—serve as a robust foundation for analyzing human interaction with each other and the world. However, the pervasive nature of digital technologies necessitates the inclusion of new dimensions that account for the complexities of contemporary life. The proposed postdigital axe bridges these gaps, offering a framework for understanding how our experiences and interactions are being (re)shaped by the entanglement of analog, digital, and hybrid realities. The integration of a postdigital axe of resonance into Rosa’s (2019) theory of resonance and alienation contributes to postdigital and postphenomenological research in investigating ‘the complexity of a technological environment and its impacts on the body, self/identity and environment itself’ (Giambastiani 2021).

Consider the individual in the café discussed earlier, immersed in multiple layers of presence—each one a distinct yet interconnected dimension of their existence. This scenario exemplifies the multifaceted nature of our postdigital lives, in which each layer of interaction contributes to a complex web of connectivity. Yet, as the individual toggles between engagements, from academic classes and virtual gaming to real-time social interactions and sensory experiences, we must question implications for their sense of connection and fulfillment. Are these interactions genuinely resonant, or do they contribute to a pervasive sense of fragmentation and disconnection?

This paper underscores the importance of expanding resonance theory to include a postdigital axe to offer a critical lens for examining how our interactions, perceptions, and relationships are mediated by digital technologies. By bridging Rosa’s (2019) theory with postdigital research, we can foster a more nuanced and unified understanding of contemporary human experience. This synthesis advances theoretical discourse and has practical implications for shaping policies, practices, and educational approaches in an increasingly postdigital world. As we continue to grapple with the implications of our postdigital existence, further research into these expanded axes of resonance will be crucial.

## Footnotes

1 An animated figure illustrating the concept of ‘co-presence’ can be found at: https://doi.org/10.6084/m9.figshare.26049511.
